# An Investigation into Sheet-Inconel 718 Forming with Flexible and Metal Tools—Simulation and Experiment

**DOI:** 10.3390/ma17133168

**Published:** 2024-06-28

**Authors:** Maciej Balcerzak, Stanislav Rusz, Radek Čada, Martin Pastrňák, Ondřej Hilšer, Miroslav Greger

**Affiliations:** 1 Department of Metal Working and Physical Metallurgy of Non-Ferrous Metals, Faculty of Non-Ferrous Metals, AGH University of Krakow, al. Adama Mickiewicza 30, 30-059 Cracow, Poland; 2 Department of Mechanical Technology, Faculty of Mechanical Engineering, VSB—Technical University of Ostrava, 17. Listopadu 2172/15, 708 00 Ostrava, Czech Republic; stanislav.rusz@vsb.cz (S.R.); radek.cada@vsb.cz (R.Č.); martin.pastrnak@vsb.cz (M.P.); ondrej.hilser@vsb.cz (O.H.); gremiger@seznam.cz (M.G.)

**Keywords:** elastomeric punches, metallic dies, Inconel 718, numerical simulation, sheet metal forming, 3D optical scanning

## Abstract

The article presents the results of numerical simulations and experimental tests of plastic forming sheets made from the difficult-to-deform nickel alloy Inconel 718 with a thickness of 1 mm, using punches made from elastomeric materials with hardness 50–90 Shore A and steel dies. Elastomeric stamps were created in the form of five layers with a diameter of 160 mm. The influence of the hardness of the elastomeric punches on the geometry of the elements obtained was determined. The dies were made from 90MnCrV8 steel with a hardness of over 60 HRC. Their task was to obtain the expected shape of the element while generating various stress states in specific areas of the semi-finished product. The research was carried out using an original device whose operating principle was based on the Guerin method. The shape and dimensions of the elements made from Inconel 718 nickel alloy were determined by optical 3D scanning. The geometry of the drawpiece showed a significant impact of the hardness of the layered elastomer matrices on the degree of shape reproduction. The results obtained from numerical modeling were confirmed by the results of experimental tests. It has been shown that the hardness of the elastomeric material used for punches for plastic forming Inconel 718 nickel alloy sheets should be adapted to the shape of the drawpiece. It was also found that one of the important aspects of plastic forming sheets using the Guerin method is the tendency to obtain a diversified shape of the final elements.

## 1. Introduction

For many years, the aviation industry has extensively used sheets crafted from challenging-to-deform alloys. Research has focused on the mechanical properties and microstructure of these materials [1]. One example is the analysis of the forming limit diagram of AMS 5599 sheet metal [2]. The coupled thermomechanical responses of nickel-based superalloys during deformation have also been measured [3]. Additionally, statistical analysis and optimization of the shear-spinning process have been conducted [4], and innovative applications of electromagnetic forming have been explored [5].

Alongside traditional techniques, electromagnetic forming methods [6] and those employing adaptable tools [7] have found application. This arises from the necessity to manufacture numerous components with diverse geometries in tightly restricted quantities. Aircraft design requires numerous components that meet meticulous standards’ criteria concerning dimensional precision and strength [8]. Elastomeric materials are used most frequently for plastic-shaping aluminum sheets [9,10] and aluminum alloys [11]. However, the authors’ reports show that materials that are difficult to deform can be effectively shaped, such as Fe alloys of high strength [12], Ti alloys [13], or Ni-based superalloys [14,15]. The plastic shaping of logs from these alloys using elastomeric materials involves many challenges. The main limitation is the inability to shape sheets at elevated and high temperatures, resulting from the limited resistance to the temperature of the elastomer [16,17]. In addition, obtaining elements with limited edge-rounding radii turns out to be a challenge due to the spring-back phenomenon [18,19,20] and the limited ability to deform the sheet metal [21,22]. In sheet metal forming (SMF) processes using punches made of flexible materials, dies are used, among others, steel, aluminum, wood, or plastics. The choice of matrix material depends on many factors, including the designer’s knowledge, required properties, and economic considerations. In industry, in processes using elastomeric materials for stamping [23], aluminum dies are often used due to their excellent strength-to-weight ratio. This is an important aspect due to the manual placement of dies in the press working area. However, it must be clearly stated that steel dies are characterized by high strength and abrasion resistance. Due to the high costs of tool production, steel dies are used in mass production [24,25].

Elastomeric materials are part of the category of cross-linked amorphous polymers, capable of undergoing deformations of up to 600%. In addition, they exhibit favorable shape-memory properties [26,27]. Among the fundamental materials within the elastomer group are natural rubber, synthetic rubbers, silicones, and polyurethanes [28,29,30,31]. Polyurethane stands out as the preferred flexible material for SMF tools, and it is renowned for its exceptional resistance to wear, thermal stability, and chemical resistance. Crucially, polyurethane has viscoelastic properties [32], making it resemble an incompressible liquid during SMF. Consequently, when parts are formed within a sealed container, the elastomeric material applies uniform pressure to the sheet metal. Despite the widespread use of elastomeric materials in the SMF process, their efficacy in this context has still not been explored sufficiently [33,34,35]. Numerous studies [30,36,37] aimed at analyzing SMF processes using flexible punches have consistently demonstrated the significant advantage of polyurethane over alternative materials.

Analysis of the sheet metal plastic-forming process using elastomeric tools has been presented in many publications. The authors focused on computer simulations of the rubber-shaping process [38,39], stamping using a flexible punch [40,41,42], and the bending process [43]. The findings presented in these publications confirm the feasibility of achieving results closely consistent with the experimental findings. Some authors conducted research on the properties of the process or elements. For example, Ramezani et al. [28] presented results on the spring-back of sheet metal during plastic forming with flexible tools, while Ali et al. [44] studied plastic-forming processes in terms of friction and lubrication conditions. Elastomeric tools have been widely used in the rubber pad process to create microchannels [45,46], emboss [47,48], and model thin metal plates [49]. As part of the work, it was decided to select Inconel 718 nickel alloy sheets, used, among others, in the aviation industry, intended for plastic shaping using layered punches made of elastomeric materials due to the high strength of the sheets and their difficulty in deformation with metal tools. Shaping products made of high-strength sheet metal requires the elastomeric material to be subjected to high pressure, which is necessary when forming elements from such materials. The information contained in various reports and publications from aviation industry specialists shows that there are no comprehensive guidelines on the type and properties of elastomeric materials used in the SMF of difficult-to-deform materials, which was the motivation to carry out the research, the results of which are presented in this article. Many researchers have conducted research on the sheet metal-forming process using elastomeric materials. However, they focused mostly on the use of one hardness variant of the elastomeric material and on metal alloys that were relatively easy to deform.

This publication is a continuation of research work on the deformability of Inconel 625 sheets, the results of which were presented in publication [50]. The aim of the research is to determine the influence of the mechanical properties and hardness of elastomer punches and steel dies on the possibility of the plastic shaping of Inconel 718 alloy sheets. Layered elastomer punches with Shore A hardness (ShA) ranging from 50 to 90 were used for forming. A specially made 90MnCrV8 steel die allowed the assessment of the stress state in the formed element and its impact on the quality of stampings produced using a method similar to Guerin’s [34].

## 2. Methods and Materials

### 2.1. Materials Subjected to Testing

#### 2.1.1. Metallic Material Undergoing Forming

**Figure 1:**
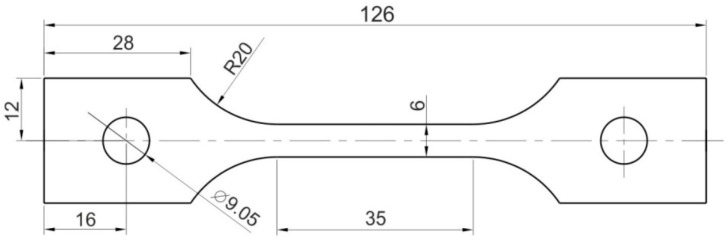
Shape and dimensions of the specimens used for mechanical properties testing.

In the case of the presented tests, sheets of nickel alloy Inconel 718 with a thickness of 1 mm were used. A uniaxial tensile test, conducted according to the guidelines outlined in standard EN ISO 6892-1:2020-05 [51], was used to assess basic mechanical properties. The shape and dimensions of the specimens used are depicted in Figure 1.

To determine the strength properties, a uniaxial tensile test was conducted using the Z100 materials testing machine (Zwick/Roell, Ulm, Germany). A strain rate of 0.008 s^−1^ was applied. The results of the tests allowed for the determination of the mechanical parameters of the Inconel 718 sheets of the nickel alloy tested, including the ultimate tensile strength *R*_m_, the yield strength *R*_p0.2_, and the elongation *A*. Additional tests were also performed to assess material anisotropy. The samples were cut at angles of 90°, 45°, and 0° relative to the rolling direction of the tested sheet. The elongation of the samples for anisotropy testing was set at 25%, a value determined based on analyses performed during tensile tests until fracture occurred. Uniaxial tensile tests were conducted using 3 samples for each test variant.

**Figure 2:**
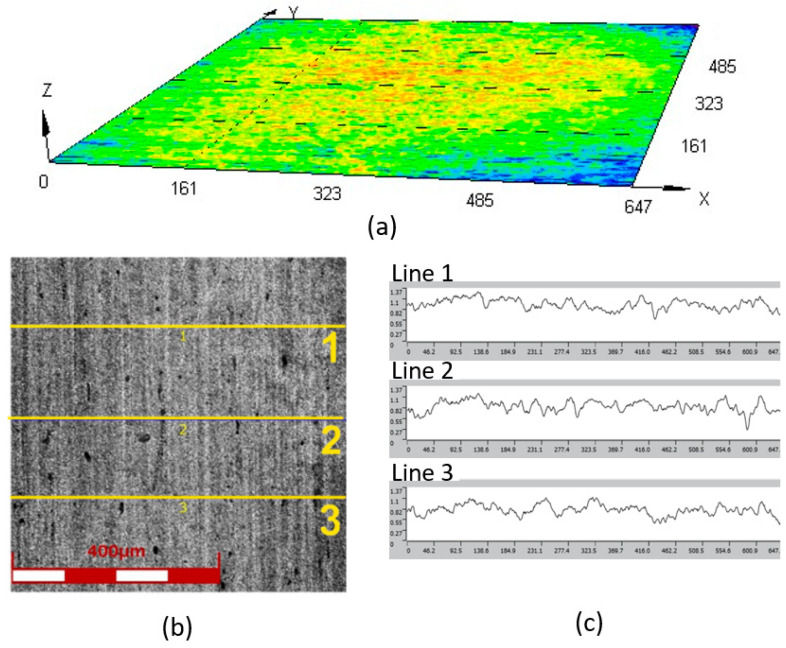
(**a**) Topography of the examined surface fragment, (**b**) surface fragment of the Inconel 718 alloy sheet undergoing roughness measurements, and (**c**) linear roughness profiles.

The surface quality was analyzed using a digital laser microscope, the LEXT OLS4100 (Olympus, Tokyo, Japan), which allowed for non-contact surface observation of elements and determination of the main roughness parameters of individual areas on the surface of the tested elements, including sheets made of the Inconel 718 alloy and elements made of polyurethane with varying hardness. Using the 3D measurement capabilities with a resolution of 10 nm, topography maps of the surface of the samples subjected to testing were developed. To enhance the accuracy of the results, basic roughness parameters such as the average roughness of the surface *R*_a_ and the height of the ten largest irregularities *R*_z_ were calculated on the basis of three measurements performed on each sample. The average results for the surface roughness measurements were 0.11 μm for *R*_a_ and 0.74 μm for *R*_z_. Standard deviation Figure 2 shows the topography map of the surface, as well as linear roughness profiles for samples made of the Inconel 718 alloy. The tests were conducted using 3 independent measurement lines.

#### 2.1.2. Material of Inserts

The research used polyurethane materials dedicated to sheet-forming processes with hardness ranging from 90 to 50 ShA. All elements tested, including samples and target-forming inserts, were manufactured in a single production batch to avoid differences resulting from variable properties of materials from different production batches. Hardness and uniaxial compression tests were conducted using samples to characterize the strength properties of the materials.

Uniaxial compression tests were performed according to the guidelines outlined in the ASTM D575-91 standard [52]. Dedicated samples with a height of 13 mm and a diameter of 28.5 mm were used. The deformation speed of the tested samples was 12 mm/min. Compression was performed until the sample height reached 10 mm. No lubricant was applied during the tests. Three independent measurements were made for each hardness variant of the polyurethane sample. The averaged surface roughness results for five different hardness variants of the elastomeric inserts are presented in Table 1. The tests were conducted using 3 independent measurement lines.

### 2.2. Testing the Wear Resistance of Polyurethane Components

**Figure 3:**
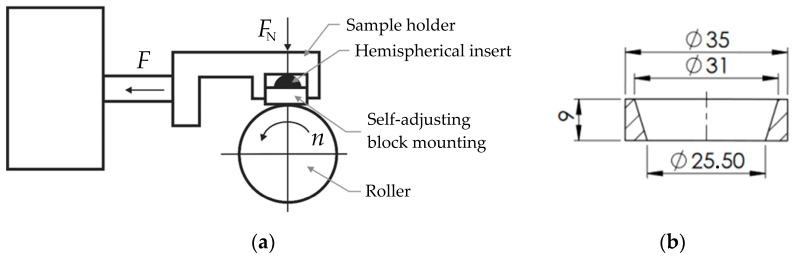
(**a**) Operating principle of the device to measure abrasive wear of materials, and (**b**) shape and dimensions of the test elements made of polyurethane.

Tests were carried out to determine the wear resistance of elastomeric materials with hardness ranging from 90 ShA to 50 ShA in abrasive contact with a counterpart made of nickel alloy Inconel 718. A T-05 roller-block tester (Institute of Precision Mechanics, Radom, Poland) was used for the tests. Since the forming process was conducted at room temperature, wear resistance measurements were also performed at standard room temperature. No lubricants were applied. Wear resistance tests were conducted for 3 sets of samples and countersamples for each measured variant. The operational principle of the device is illustrated in Figure 3a. The dimensions of the elastomeric material samples are shown in Figure 3b.

The measurements were carried out under constant conditions, with the rotational speed of the polyurethane ring set at *n* = 136 rpm and a compressive force of *F_N_* = 50 N. The test was carried out with a constant distance for all samples, set at 150 m. To determine the coefficient of friction, denoted as *µ* in the equation, the friction force, which varied during the duration of the test, was recorded. The coefficient of friction was determined according to Equation (1).

(1) $$\mu =\frac{F}{F_{N}}$$

The coefficient of friction was presented as the value calculated based on the friction path considered in the study. The mass loss expressed as a percentage (Δ*m_cs_*) of the counterpart made of elastomeric material was calculated using Equation (2).

(2) $${∆m}_{cs}=\frac{m_{p}-m_{k}}{m_{p}}\times 100\%$$

The same methodology for measuring mass loss was applied to the samples made of the nickel alloy Inconel 718. In the equation, the initial mass of the sample was denoted as mp, while the mass of the sample after the tests was denoted as *m_k_*.

### 2.3. Methodology of Numerical Simulations

Numerical simulations of the forming process for Inconel 718 sheets were conducted using the Impetus Afea (Impetusafea AB, Huddinge, Sweden) software based on finite element methods. The mechanical properties of Inconel 718 alloy sheets were developed on the basis of dedicated uniaxial tensile tests utilizing specialized samples. Uniaxial compression tests of elastomeric materials were used to determine the material coefficients used in the numerical simulations. This was described in detail in Section 2.1.2. Cylindrical samples made of polyurethane with various hardnesses were used. A two-parameter Mooney–Rivlin constitutive model was used.

(3) $$\sigma_{1}=\frac{2C_{1}}{3}\left(2\lambda_{1}-\lambda_{2}-\lambda_{3}\right)-\frac{2C_{2}}{3}\left(\frac{2}{\lambda_{1}}-\frac{1}{\lambda_{2}}-\frac{1}{\lambda_{3}}\right)-\rho$$

(4) $$\sigma_{2}=\frac{2C_{1}}{3}\left(2\lambda_{2}-\lambda_{3}-\lambda_{1}\right)-\frac{2C_{2}}{3}\left(\frac{2}{\lambda_{2}}-\frac{1}{\lambda_{3}}-\frac{1}{\lambda_{1}}\right)-\rho$$

(5) $$\sigma_{3}=\frac{2C_{1}}{3}\left(2\lambda_{3}-\lambda_{1}-\lambda_{2}\right)-\frac{2C_{2}}{3}\left(\frac{2}{\lambda_{3}}-\frac{1}{\lambda_{1}}-\frac{1}{\lambda_{2}}\right)-\rho$$

(6) $$\rho =-K\varepsilon_{v}$$

In the selected constitutive model, the stresses *σ*_1_, *σ*_2_, *σ*_3_ correspond to principal stresses, while *λ*_1_, *λ*_2_, *λ*_3_ represent the eigenvalues of the Cauchy–Green stretch tensor. The pressure *p* is described as a linear function of volumetric strain *εᵥ*, where *C*_1_, *C*_2_, and *K* represent material constants determined based on the tests carried out.

**Figure 4:**
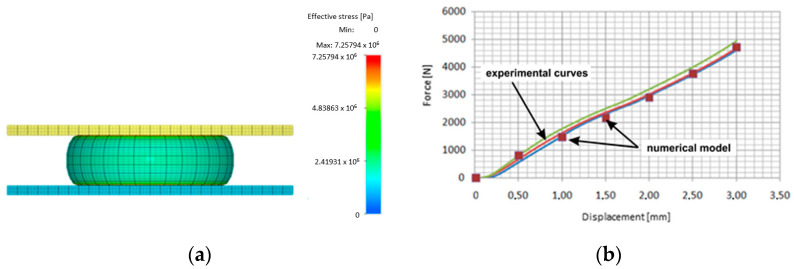
(**a**) Results from numerical computations of the uniaxial compression process of a cylindrical sample and (**b**) fitting of the experimental curve to the result obtained during numerical simulations.

As a result of the high accuracy and availability of the software used, the Mooney–Rivlin material model was chosen for numerical simulations of the elastomeric material. A geometric representation of the conducted tests was prepared, taking into account the shape of the samples. Experimental tests and numerical simulations of the tests were carried out to determine the material coefficients *C*_1_ and *C*_2_. Figure 4 shows the results of the numerical simulations of the uniaxial compression process of the sample and the fitting of the curve obtained from the numerical simulations with the curve obtained during the experiment. Table 2 presents the values of the coefficients *C*_1_, *C*_2_, and *K* for all hardness levels of the elastomeric materials subjected to tests.

For numerical simulations, a constant friction coefficient of *μ* = 0.09 was established to facilitate the comparison of the forming results for elastomeric inserts of varying hardness. Surface elements were applied to the die, punch, and container because of their treatment as rigid bodies. The sheet was made of Inconel 718 alloy, and the elastomeric inserts were treated as deformable. The calculations used the von Mises criterion, the elastic–plastic principle of plastic flow, and an explicit time integration scheme. A penalty function was used for the contact algorithm.

**Figure 5:**
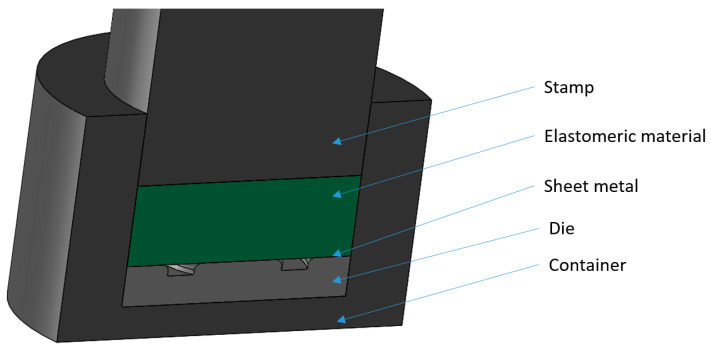
Simplified 3D model of the tool for forming the Inconel 718 alloy sheet using elastomeric materials, used for conducting numerical simulations.

Numerical simulations were conducted using a simplified 3D model developed based on the shapes of real tools. The decision to simplify the process was made to reduce the computation times required to obtain results due to the lack of a significant influence of the additional elements necessary in the forming process, such as fixtures, guiding systems for mounting plates, etc. Figure 5 illustrates the simplified 3D model used for performing numerical simulations.

**Figure 6:**
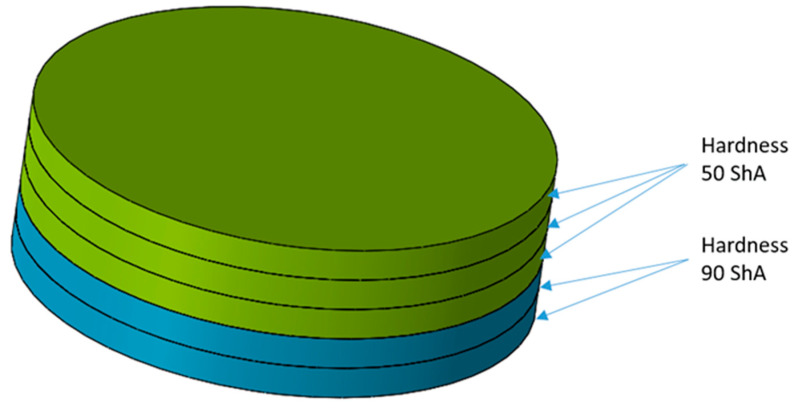
Example of a hybrid set of polyurethane inserts using two different hardness levels.

**Figure 7:**
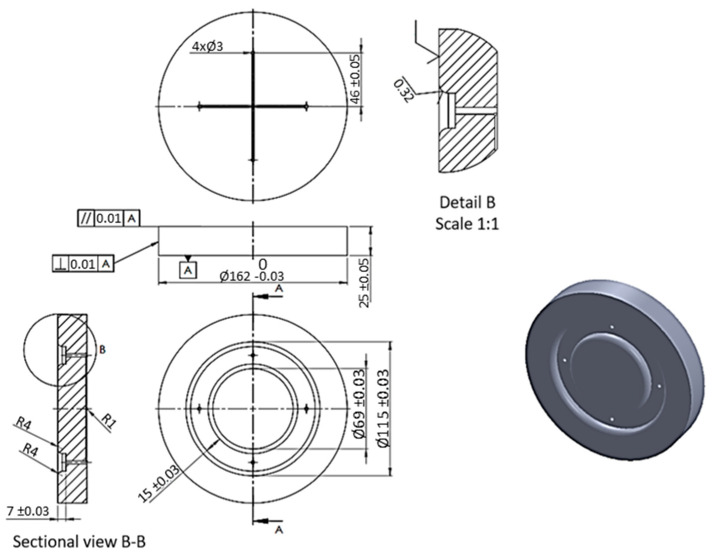
Dimensions of the forming tool used in the process for forming sheets of Inconel 718 alloy.

The numerical simulations were divided into two stages. The first stage involved the use of a set of five elastomeric inserts of the same hardness. The second stage involved the use of two different hardness levels of elastomeric inserts in one tool. Extreme hardness levels were used for investigation. Sets containing 2 inserts of 50 ShA hardness and 3 inserts of 90 ShA hardness were examined, as well as the reverse variant where 2 inserts of 90 ShA hardness and 3 inserts of 50 ShA hardness were used. The most significant difference in the case of hybrid sets was the hardness of the elastomeric material directly in contact with the surface of the formed sheet. In the first case, it was 50 ShA, while in the second case, it was 90 ShA. An example of such a set of elastomeric inserts is shown in Figure 6. The total thickness of the entire set of polyurethane inserts amounted to 50 mm, divided into 5 individual inserts, each with a thickness of 10 mm. The diameter of the elastomeric elements was 160 mm. The die responsible for shaping the elements was made of 90MnCrV8 steel. Its shape is depicted in Figure 7.

**Figure 8:**
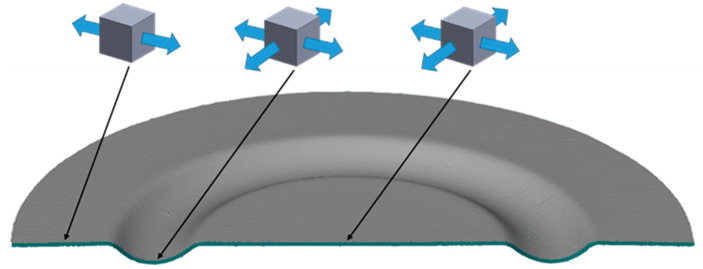
Cross-sectional view of the 3D model of the element with stress states applied in specific areas.

The die was designed to showcase the influence of the hardness of the elastomeric inserts on the quality of the elements produced. The possibility of making an oval indentation using different hardness levels of polyurethane inserts was tested. Technological tilts and radii of curvature were not applied in the lower part of the recess to prevent the complete forming of the prepared shape. This approach was designed to allow the determination of differences in the forming depth for various hardness levels of the inserts. Tensile stresses were designed to occur in the outer part of the element, with minor compressive stresses resulting from the circular shape of the obtained element. In the formed area, a flat stress state predominates, with tensile stresses being dominant. Stress distributions are depicted in Figure 8.

### 2.4. Forming Procedure

**Figure 9:**
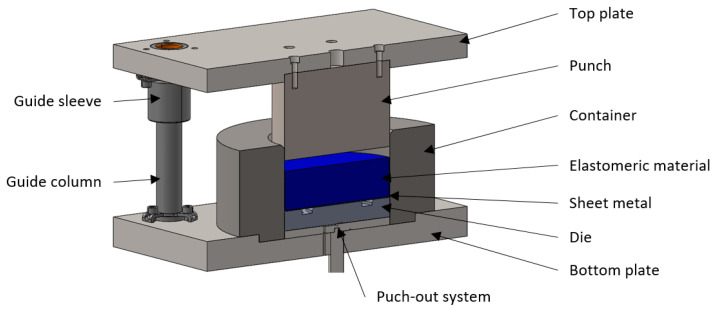
3D model of the modified device for sheet metal forming using elastomeric materials.

For experimental testing of the sheet-forming process with Inconel 718 nickel alloy, a tool designed based on the Guerin method was used. This method is applied to form elements in the aerospace and automotive industries for small production batches. The device was modified because of the need for frequent replacement of polyurethane-forming elements. The developed ejection system allowed for quick replacement of the forming set containing elastomeric inserts, the die, and the formed sheet. The assumptions obtained during the numerical simulations were tested using the Hydromega 150T hydraulic press (Hydromega, Gdynia, Poland), which has a maximum pressing force of 1500 kN. A detailed 3D model of the device containing all necessary components is presented in Figure 9.

**Figure 10:**
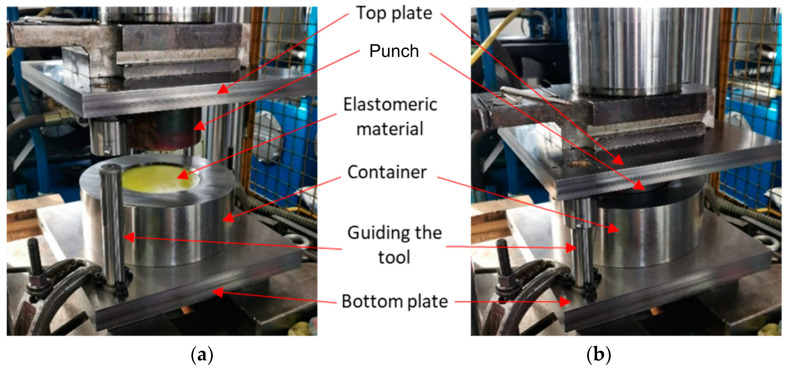
Toolset prepared for the forming process (**a**) mounted on the press and (**b**) the toolset during the forming process.

In Figure 10, the toolset is presented in two phases of the process. Figure 10a shows the tool prepared for the process, while Figure 10b shows the element-forming stage. All tool components were attached to the upper and lower plates, which were subsequently mounted to the upper and lower parts of the press worktable. The ejection system relied on a cylinder mounted under the lower part of the press worktable. When the ejection system was extended, the die, sheet undergoing the forming process, and set of elastomeric inserts were placed on its base. The phasing of the upper part of the container is aimed at the proper alignment of the elements relative to the center of the tool. Upon activating the press movement, force was exerted on the elastomeric material, which, under pressure, deformed the sheet of Inconel 718 nickel alloy and forced it to adopt the shape of the rigid die made of steel. During forming, H-336 grease (Molydal, Saint-Maximin, France) was used. Its application was necessary due to the high coefficient of friction, which adversely affected the quality of the formed elements. The reduction in friction differences resulting from the hardness of the elastomeric inserts positively influenced the comparability of the results obtained.

### 2.5. Methodology for Evaluating the Formed Parts Obtained Using a 3D Optical Scanning Process

**Figure 11:**
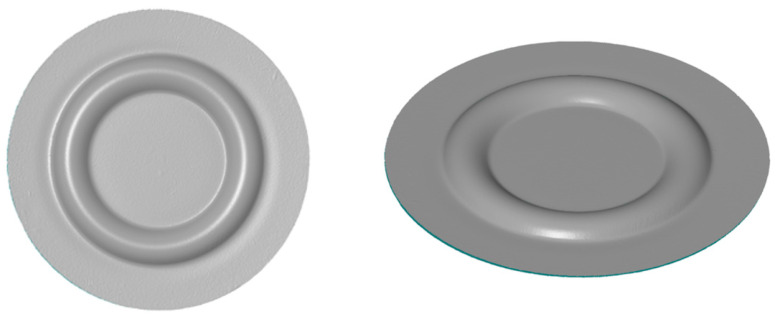
Example 3D model obtained in the 3D scanning process.

For the dimensional conformity assessment of the parts produced, a 3D scanner Atos Core 200 (Carl Zeiss GOM Metrology GmbH, Braunschweig, Germany) was used. Thanks to the measurements, 3D models of all variants obtained with an accuracy of 0.017 mm were created. They were subjected to dimensional and comparative analysis to verify the influence of the hardness of the elastomeric inserts on the geometric fidelity of the formed parts. An exemplary 3D model under analysis is depicted in Figure 11.

The key measurement value used to compare the formed elements was determined as the forming depth. This measurement was carried out by determining the maximum distance between the formed indentation and the reference plane located on the surface of the element. Using the obtained 3D models, surface flatness, diameter change in the formed element, forming uniformity, and sheet thickness change were measured.

**Figure 12:**
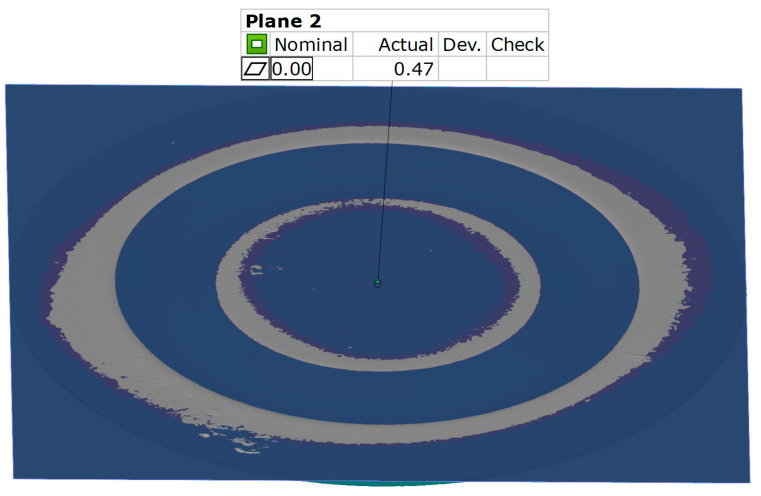
Result of the surface flatness measurement.

To determine the flatness of the surface of the formed element, the best-fit function was used. Two parallel planes are created to define the extreme areas on the surface of the element. The value presented is the distance between the two planes created. An example result of the surface flatness measurement is shown in Figure 12.

**Figure 13:**
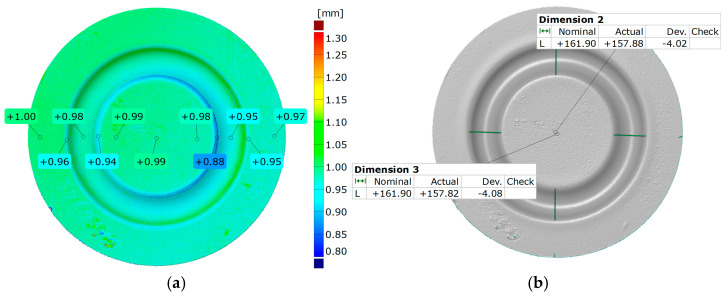
Example measurement result of (**a**) thickness of the formed element and (**b**) diameter change in the formed element.

The process of generating results for thickness alterations (Figure 13a) can be conducted by establishing the normal orientation of the surfaces examined. This is achieved by assessing the difference between the model surfaces at each recorded point. To assess the diameter change, the manual measurement of distances was used (Figure 13b). Measurements were carried out parallel to and perpendicular to the reference point. The data obtained were presented numerically, serving as a benchmark value derived from the difference between the initial diameter of the element undergoing the forming process and the measured value after the process.

**Figure 14:**
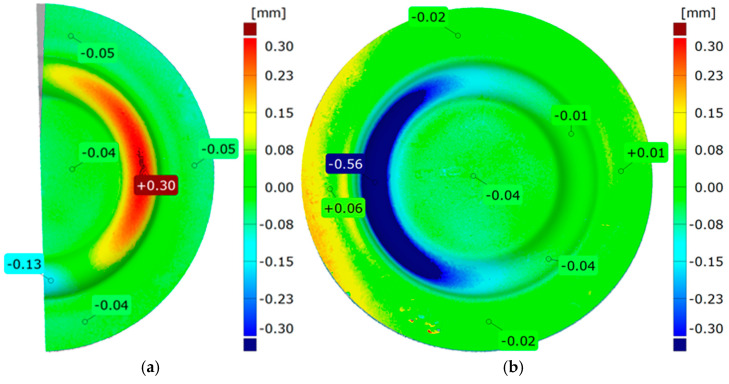
Results for (**a**) uniform forming and (**b**) comparison of forming depths for different hardness levels of elastomeric inserts.

The assessment process of forming uniformity was carried out by dividing the 3D model of the formed part into two sections. They were then overlaid using the best-fit function. A color-coded deviation map was used to present the results, with local deviation measurements applied in key areas. The comparison of forming uniformity is shown in Figure 14a. In addition, measurements were performed to determine the influence of the hardness of the elastomeric inserts on the forming outcome. Using the initial indentation areas and the flat surface of the element, scans of parts made with different hardness levels of the elastomeric inserts were overlaid. The best-fit function of the models was chosen to enhance measurement accuracy. The results of the comparison are presented in Figure 14b.

## 3. Results and Discussion

### 3.1. Results of Inconel 718 Sheet—Mechanical Properties

**Figure 15:**
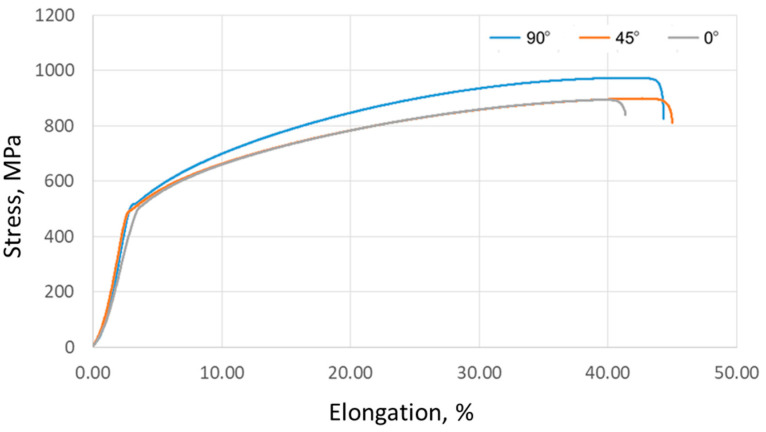
Results of Inconel 718 sheet—tensile curves.

Figure 15 shows the tension curves of Inconel 718 sheets, which show three different data series corresponding to samples cut at angles of 0°, 45°, and 90° relative to the rolling direction of the sheet. A concise overview of the fundamental mechanical parameters and anisotropy coefficients is provided in Table 3 and Table 4, respectively. These results were averaged from three separate replicates. In particular, the results of the tensile test exhibited discrepancies according to the orientation of the sample. The maximum ultimate tensile strength (973.5 MPa) was recorded for a sample aligned perpendicular to the sheet rolling direction. The range of results based on the sampling direction amounts to 79.3 MPa for tensile strength, 16.8 MPa for yield stress, and 3.6% for elongation.

### 3.2. Results of Elastomeric Materials—Mechanical Properties

The hardness of the elastomeric materials was verified through five measurements carried out for each type of elastomer used. Table 5 presents both the individual measurements and the average hardness obtained. For materials with nominal hardness values of 900, 80, and 50 ShA, the results suggest minor variations depending on the measurement site, with differences not exceeding 2 ShA. This indicates a uniform hardness across the polyurethane materials. However, for materials with an expected hardness of 70 ShA, there was a notable disparity, with the average value closely aligning with a hardness of 60 ShA.

**Figure 16:**
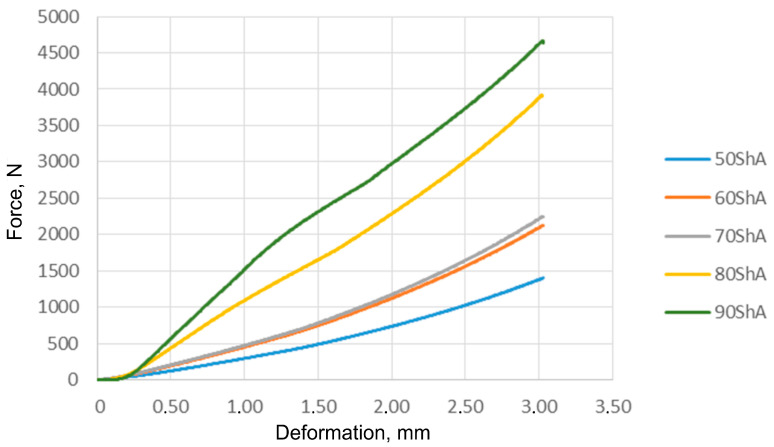
Results of elastomeric samples—uniaxial compression test.

The results of the compression tests for all examined elastomer hardnesses are shown in Figure 16. These findings served as the basis for establishing the material constants within the Mooney–Rivlin material model. The compression results indicate a pattern of improved compressive strength corresponding to increased hardness of the elastomeric material. In particular, samples rated at 70 ShA and 60 ShA exhibited a marginal variance in the results derived from the uniaxial compression tests.

### 3.3. Results of Elastomeric Materials—Wear Resistance

Using elastomeric materials with significantly lower hardness to form sheets made of difficult-to-deform alloys results in increased wear, as highlighted by previous studies [23,45]. Therefore, quantifying the wear experienced by the contact pair is essential to establish ideal parameters in the forming process. Table 6 presents the wear results obtained from testing elastomer and Inconel 718 nickel alloy samples. An observed phenomenon among the tested elastomers was the deformation of their surface upon contact with the sample, which occurred under an applied force of *F*_N_ = 50 N. This deformation directly affected the extent of the tribological contact area within the friction interface. Polyurethanes, serving as a countersample, experienced mass losses within the friction node. However, certain samples made of nickel alloy exhibited an increase in mass as a result of the transfer of material from the countersample. The coefficient of friction (COF) of the tested elastomers varied from 0.240 to 0.683. Specifically, the elastomer with a hardness of 90 ShA exhibited the smallest average COF value. The recorded COF value of 0.240 is relatively high compared to other polymer materials.

### 3.4. Results of Forming Process—Numerical Simulations

**Figure 17:**
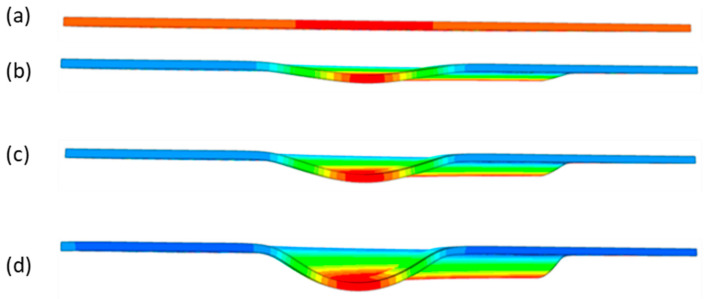
Results of modeling the plastic forming process of Inconel 718 sheet metal with elastomeric punch—(**a**) 20%, (**b**) 40%, (**c**) 60%, and (**d**) 90% of the simulation time.

During the virtual experiment, a force of 400 kN was exerted on the elastomer punch surface. Figure 17 illustrates the progressive deformation of the workpiece throughout the simulation stages. Table 7 describes the results of the simulation for different polyurethane insert setups. The data in this table are depicted in perpendicular and transverse perspectives, facilitating the evaluation of the forming results. A dedicated column displays the measured maximum embossment depth, a crucial parameter for comparing the efficacy of various polyurethane insert configurations. The forming depth was prioritized as the primary numerical output because of its straightforward validation against experimental results. Among the configurations tested, hybrid setups with different hardnesses of polyurethane exhibited the highest sheet metal deformation in the embossed areas (5.398 and 5.113 mm). In particular, inserts with lower hardness in contact with the sheet surface yielded greater deformation in the embossments.

**Figure 18:**
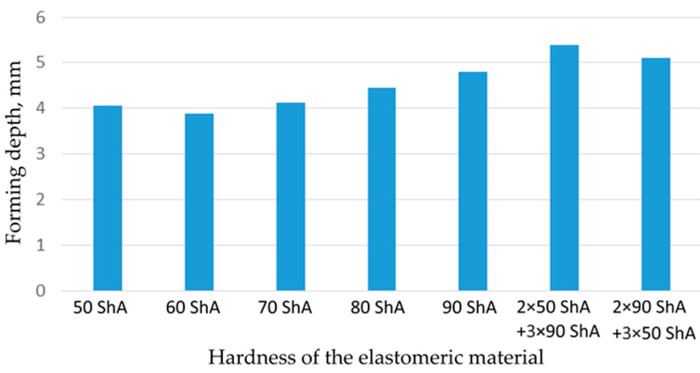
Results of the influence of different hardness of elastomer punches on the maximum depth of the cups.

**Figure 19:**
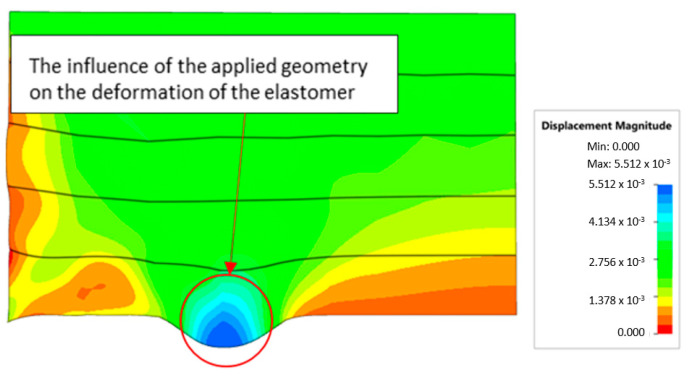
Deformation of a layered elastomeric punch with different hardness 2 × 50 ShA + 3 × 90 ShA.

Figure 18 illustrates a comparison of the maximum emboss depth in various polyurethane hardness options. The die shape was specifically tailored to assess the sheet metal forming process using a cylindrical geometry in order to scrutinize the effect of design on the forming precision. When 5-layer inserts were used with identical hardness, increasing the hardness of a polyurethane insert correlated with increased material deformation in embossed areas (as shown in Figure 18). Meanwhile, Figure 19 shows the impact of tool geometry on the deformation of the elastomeric material.

The most profound embossments were achieved with configurations featuring layered polyurethane inserts composed of two layers at 50 ShA hardness and three layers at 90 ShA hardness. These configurations yielded a depth of 5.398 mm, where two layers with 50 ShA hardness were placed directly above the workpiece surface, while three layers with 90 ShA hardness were placed above the 50 ShA inserts. On the contrary, the least favorable result, 3.883 mm, with a polyurethane tool comprising five inserts at 60 ShA hardness, was observed. This revealed a notable difference of 1.515 mm between the best and worst combinations. Specifically, using the configuration of two layers at 50 ShA hardness and three layers at 90 ShA hardness allowed a 28% deeper stamping depth compared to employing five inserts at 60 ShA hardness.

**Figure 20:**
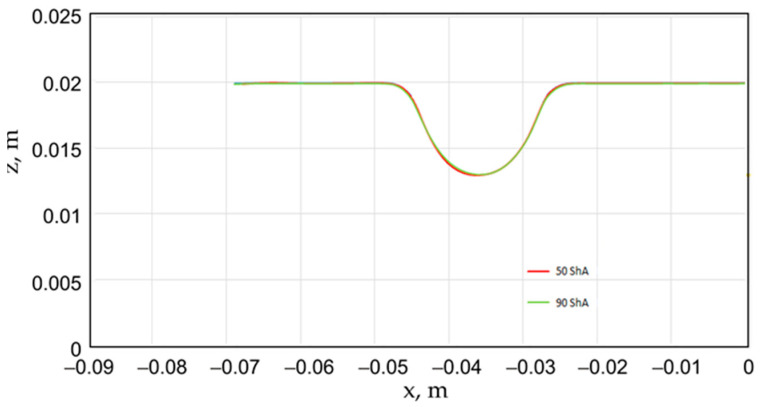
Geometry of cups for elastomer punches with hardnesses 50 ShA and 90 ShA—forming force 1000 kN.

Additionally, numerical simulations were conducted with an increase in the forming force of 1000 kN. When the forming force was increased, no significant changes were observed, depending on the hardness of the elastomeric material used. Figure 20 provides a comprehensive overview of sheet metal deformations during forming with elastomer inserts ranging from 50 to 90 ShA hardness under a pressure of 1000 kN.

Due to the absence of discernible disparities in the simulation outcomes under a forming force of 1000 kN, it was determined to rely on the results derived from a forming force of 400 kN for experimental validation. When comparing inserts of identical hardness, the shallowest emboss depth was observed for inserts rated at 60 ShA (Figure 18). However, for inserts composed of different hardness levels, the most significant emboss depth was achieved with the configuration of two layers 50 ShA and three layers 90 ShA (Figure 18).

### 3.5. Results of the Forming Process—Experimental Tests

Table 8 provides a comprehensive overview of the results obtained by forming drawpieces on a hydraulic press under a pressure of 1000 kN, showcasing the impact of different arrangements and hardness levels of polyurethane inserts. Each entry in the table includes photographs of the samples, a 3D scan image, and the measured maximum forming depth for the respective variant. In particular, the variance in the forming depth among the different configurations of the elastomer inserts analyzed amounted to 0.19 mm.

For the variant featuring five inserts with a hardness of 60 ShA, the maximum forming depth was recorded at 5.37 mm. On the contrary, for the configuration comprising two layers at 50 ShA and three layers at 90 ShA, the maximum forming depth was significantly higher, reaching 5.56 mm (Table 8).

**Figure 21:**
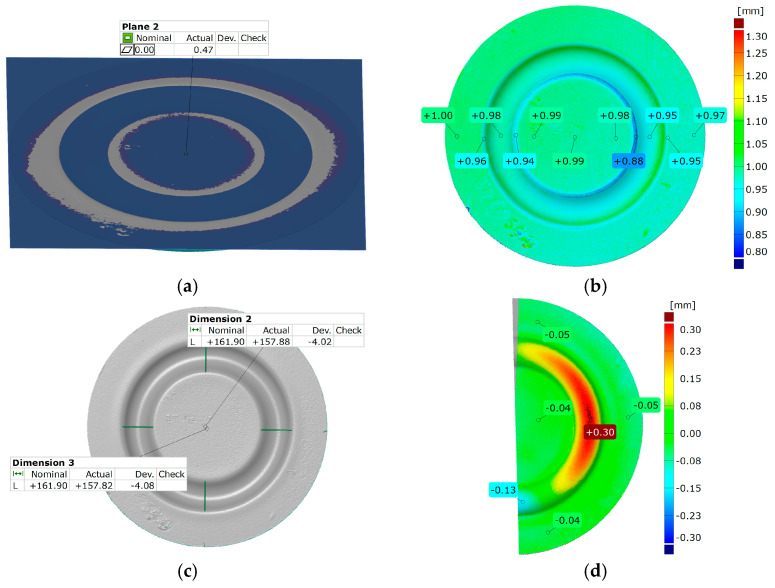
Geometry of plastic-forming cups using five layers of elastomers with a hardness of 60 ShA: (**a**) surface flatness; (**b**) thickness; (**c**) changes in the diameter of the cup; (**d**) uniformity of the forming.

**Figure 22:**
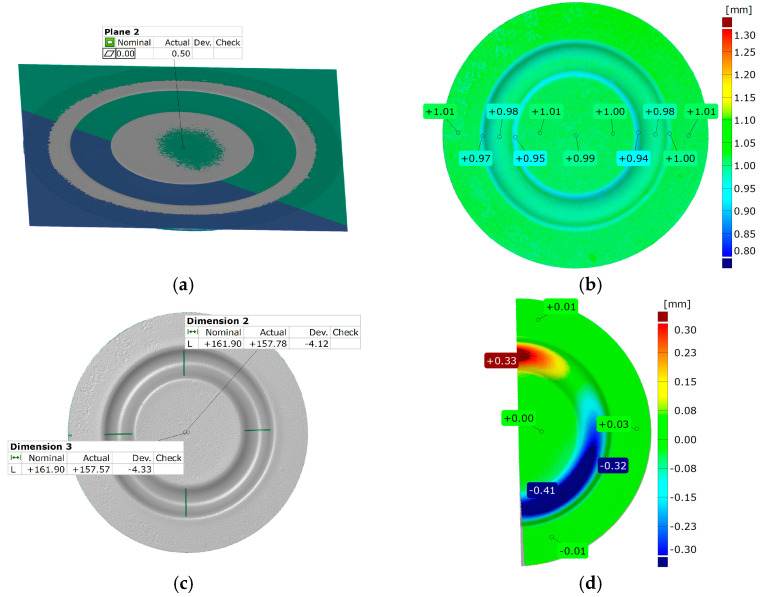
Geometry of plastic-forming cups using two layers of elastomers with a hardness of 50 ShA and three layers of elastomers with a hardness of 90 ShA: (**a**) surface flatness; (**b**) thickness; (**c**) changes in the diameter of the cup; (**d**) uniformity of the forming.

Figure 21 and Figure 22 show the results of the geometric analysis performed on the drawpieces. In Figure 21, a configuration using five inserts with a hardness of 60 ShA was used, which exhibited the poorest ability to form elements made of the Inconel 718 alloy, according to numerical analyses. On the contrary, Figure 22 shows the results under the same forming conditions, but with an arrangement comprising two inserts at 50 ShA hardness and three inserts at 90 ShA hardness, which numerically demonstrated the highest ability to form the element.

Regarding surface flatness, both variants exhibited a difference of 0.03 mm. Specifically, for the 5 × 60 ShA variant, the surface flatness was 0.47 mm, while for the 2 × 50 ShA + 3 × 90 ShA configuration, it was 0.50 mm. In terms of wall thinning, the 5 × 60 ShA system showed a maximum difference between maximum and minimum values of 0.12 mm, resulting in a maximum wall thinning of 12%. In the 2 × 50 ShA + 3 × 90 ShA configuration, a lesser and more uniform thinning was observed, with a minimum wall thickness value of 0.94 mm, translating to a wall reduction of 7%.

Furthermore, it was observed that the change in the diameter of the formed component for the 5 × 60 ShA system was −4.02 mm longitudinally and −4.08 mm in the transverse direction. These values differed slightly for the 2 × 50 ShA + 3 × 90 ShA configuration, measuring −4.12 mm longitudinally and −4.33 mm transversely. In particular, the 5 × 60 ShA system exhibited notable discrepancies in formation uniformity, with a 0.3 mm difference between the left and right sides of the formed drawpiece. The 2 × 50 ShA + 3 × 90 ShA configuration demonstrated uniformity, with a maximum difference of 0.41 mm.

**Figure 23:**
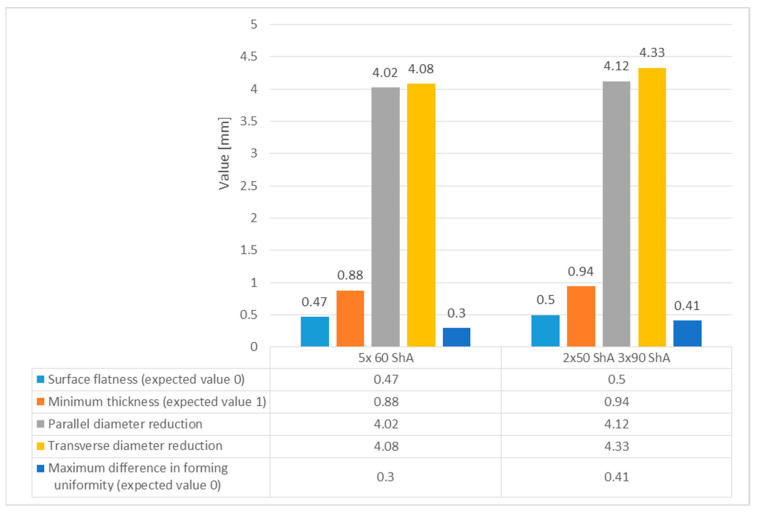
Summary of test results on the geometry of Inconel 718 sheet cups.

Figure 23 provides a tabulated summary of the measurements obtained from the formed parts.

**Figure 24:**
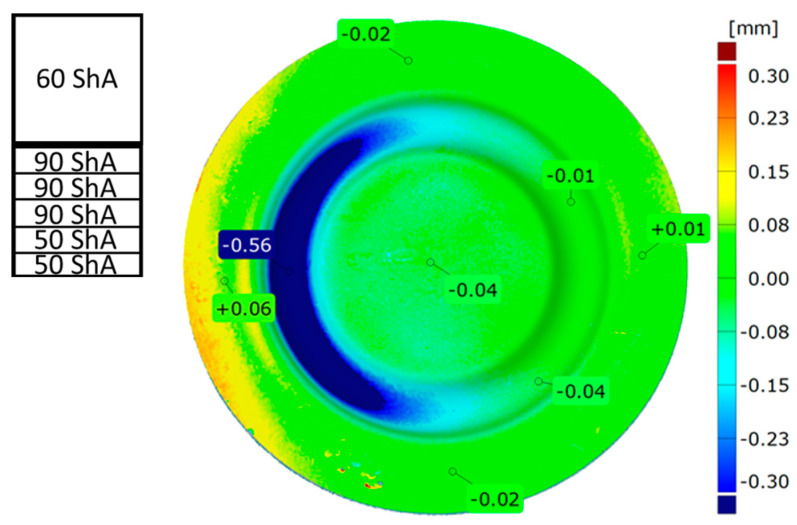
Comparison of cups made using punches with different hardnesses of elastomer layers: 5 × 60 ShA and 2 × 50 ShA + 3 × 90 ShA.

Figure 24 presents a comparative analysis of 3D scans showing measured drawpieces obtained using two different configurations of elastomer inserts. The findings substantiate a deeper emboss formation for the configuration utilizing two layers at 50 ShA hardness and three layers at 90 ShA hardness for polymeric inserts. The observed differences range from −0.56 mm to −0.01 mm on the surface of the embosses.

When the results of various combinations of inserts are scrutinized, it becomes evident that the quality of the produced element significantly varies depending on the hardness of the polyurethane material used. These results validated both numerically and experimentally, highlight the superiority of the 2 × 50 ShA + 3 × 90 ShA configuration for the main geometric parameters measured.

## 4. Conclusions

The presented publication focuses on presenting the results of numerical and experimental simulations of the sheet metal-forming process with Inconel 718 nickel alloy using elastomeric inserts ranging in hardness from 90 ShA to 50 ShA. The results demonstrate the impact of the hardness of elastomeric inserts on the quality of the obtained products and the main challenges encountered during the forming of difficult-to-deform materials using elastomeric forming elements. The most important conclusions drawn from the conducted analyses are presented below:

- The results of numerical analyses and the forming process under industrial conditions allowed us to conclude that the hardness of the elastomeric inserts should be selected based on the shape of the formed element. This modern approach, which has not been sufficiently explored yet, was based on a series of studies that utilise various die shapes for forming;
- For various levels of hardness of elastomeric inserts, a similar forming depth of approximately 5.5 mm was observed. However, there were clear differences in terms of uniformity forming and when comparing 3D models obtained in the 3D scanning process for elements shaped using different hardness levels of polyurethane inserts. An important aspect that had not been sufficiently explored previously was the determination of differences in forming uniformity. This allows us to conclude that it is one of the main problems occurring during the forming of Inconel 718 alloy sheets using elastomeric materials;
- The use of different levels of hardness of polyurethane inserts and their combinations has a significant impact on the quality of the obtained elements. By applying elastomer hardness tailored to the material being formed and the geometry of the element, better forming results can be achieved without altering process parameters. Particular attention should be paid to the significant improvement in forming quality through the use of hybrid elastomeric tools that incorporate elastomeric inserts of various hardness levels;
- The presented results demonstrate that the forming of sheets of difficult-to-deform nickel alloys using elastomeric materials is possible through the use of a standard hydraulic press. Achieving satisfactory forming results for 1 mm thick sheets made of Inconel 718 alloy during cold forming clearly indicates that this process is feasible without specialized presses dedicated to sheet forming in processes involving elastomeric materials. This is a significant achievement due to the great difficulties in forming high-strength nickel alloys. Forming such alloys using elastomeric materials could become an alternative to commonly used manufacturing processes, particularly for single components or small production runs;
- The division of the polyurethane forming set into five elements does not adversely affect the forming process. This solution allows for increased material utility in such processes as a result of increased surface wear of the elastomer in contact with the formed sheet. Frequent changes in the order of the elastomeric inserts significantly prolong the degradation process and increase the uniformity of material wear throughout the volume of the forming tool. This conclusion allows for a significant reduction in the costs of implementing this process, which further increases its attractiveness as an alternative method for forming high-strength sheets in industrial conditions.

## Figures and Tables

**Table 1:** Average values of the surface roughness parameters of elements made of polyurethane with hardness ranging from 90 to 50 ShA.

Surface Roughness Parameter	Hardness of the Polyurethane Element				
	90 ShA	80 ShA	70 ShA	60 ShA	50 ShA
*R*_a_ (μm)	0.894 ± 0.005 mm	1.228 ±0.008 mm	0.204 ± 0.004 mm	0.191 ± 0.006 mm	0.337 ± 0.008 mm
*R*_z_ (μm)	5.493 ± 0.125 mm	7.918 ± 0.154 mm	1.517 ± 0.085 mm	1.244 ± 0.063 mm	2.536 ± 0.112 mm

**Table 2:** Values of material coefficients *C*_1_, *C*_2_, and *K* for elastomeric samples of different hardness.

Hardness of the Elastomeric Sample	*K* (Pa)	*C*_1_ (Pa)	*C*_2_ (Pa)
50 ShA	4.0 × 10^9^	0.3 × 10^6^	0.15 × 10^9^
60 ShA	4.1 × 10^9^	0.59 × 10^6^	0.19 × 10^6^
70 ShA	4.2 × 10^9^	0.6 × 10^6^	0.2 × 10^6^
80 ShA	4.8 × 10^9^	1.6 × 10^6^	0.11 × 10^6^
90 ShA	4.8 × 10^9^	2.1 × 10^6^	0.1 × 10^6^

**Table 3:** Results of Inconel 718 sheet—mechanical parameters.

Sample Orientation	0°	45°	90°
Ultimate tensile stress *R*_m_ (MPa)	894.2 ± 8.4	898.9 ± 9.1	973.5 ± 7.9
Yield stress *R*_p0.2_ (MPa)	498.1 ± 5.2	497.7 ± 6.7	514.5 ± 9.1
Elongation *A* (%)	41.4 ± 2.1	45 ± 1.8	44.3 ± 2.4

**Table 4:** Results of Inconel 718 sheet—coefficients of anisotropy.

Coefficient of Planar Anisotropy	Coefficient of Normal Anisotropy
−1.08	1.21

**Table 5:** Results of elastomeric samples—hardness.

Nominal Hardness (ShA)	Hardness Measurements (ShA)					Average Hardness (ShA)
	1	2	3	4	5	
50	52	51	51	52	50	51.2
60	60	62	61	63	60	61.2
70	65	67	66	66	65	65.8
80	79	78	80	81	79	79.4
90	91	90	90	89	89	89.9

**Table 6:** Results of the elastomeric countersamples and Inconel 718 sample—wear parameters and COF.

Parameter	Elastomeric Countersample—Hardness				
	90 ShA	80 ShA	70 ShA	60 ShA	50 ShA
Weight loss of polyurethane countersample Δ*m_cs_* (%)	0.011 ± 0.002	0.43 ± 0.03	0.116 ± 0.011	0.2613 ± 0.024	0.3825 ± 0.032
Weight loss of Inconel 718 sample Δ*m*_s_ (%)	−0.008 ± 0.001	−0.064 ± 0.004	−0.0689 ± 0.006	0.0721 ± 0.004	0.0837 ± 0.002
Average COF	0.240 ± 0.012	0.683 ± 0.033	0.376 ± 0.016	0.342 ± 0.013	0.429 ± 0.021

**Table 7:** Results of the influence of different hardness of elastomer punches on the maximum depth of the cups.

Elastomeric Punch—Hardness	Results of Displacement	Maximum Cup Depth
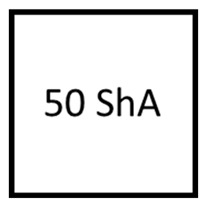	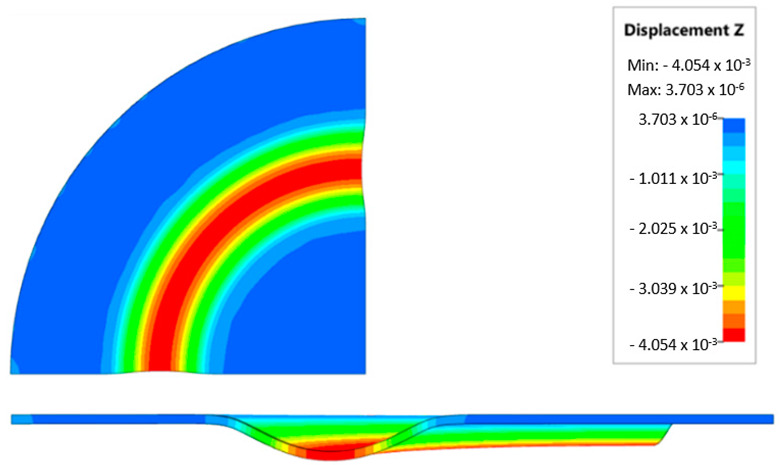	4.054 mm
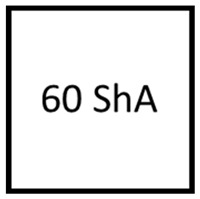	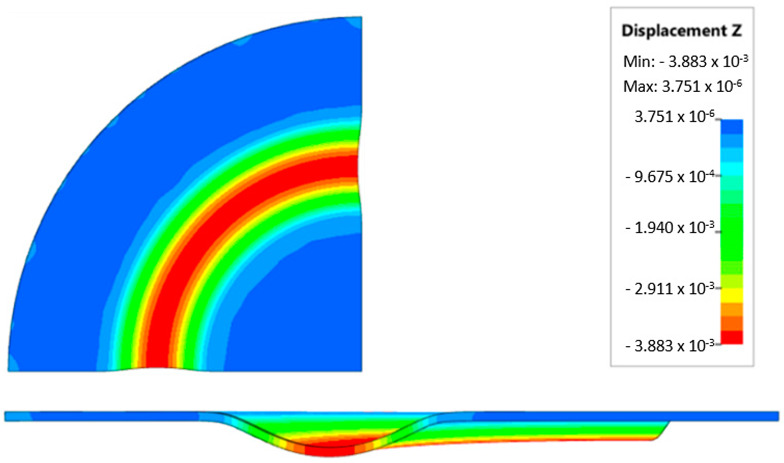	3.883 mm
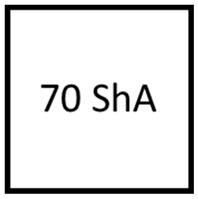	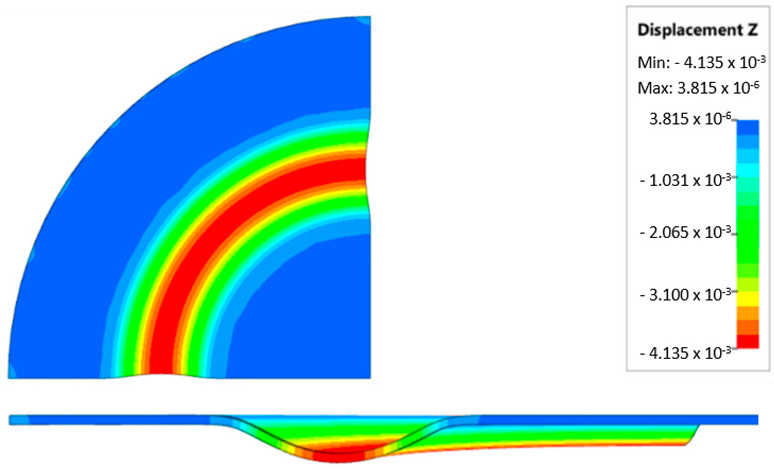	4.135 mm
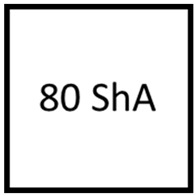	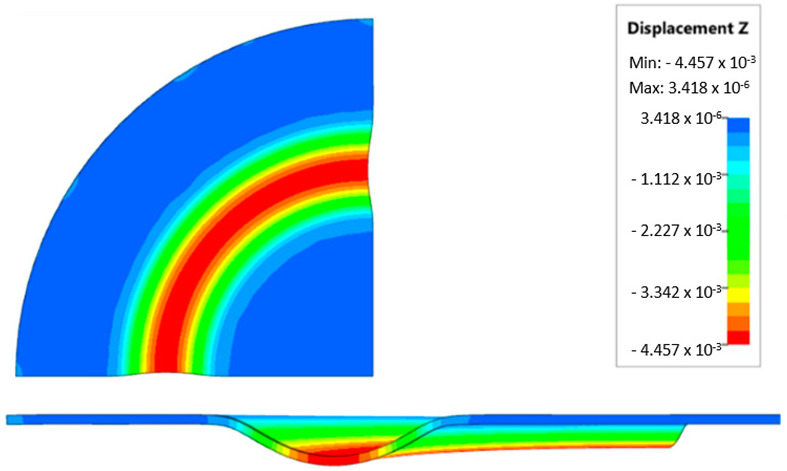	4.457 mm
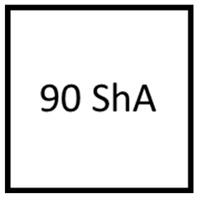	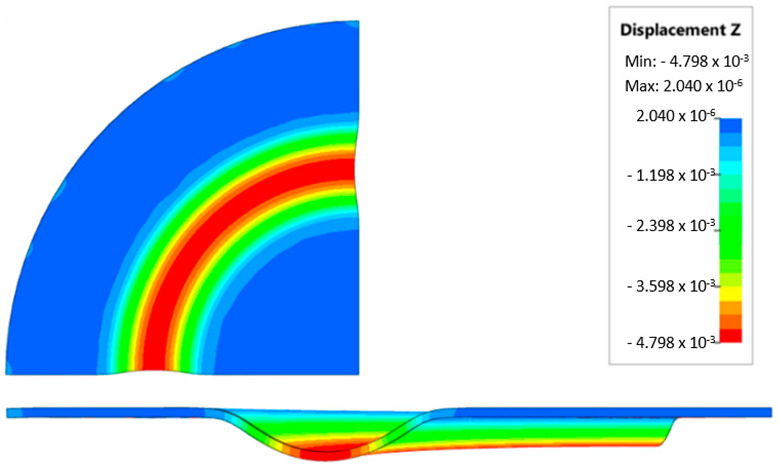	4.798 mm
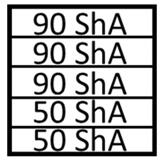	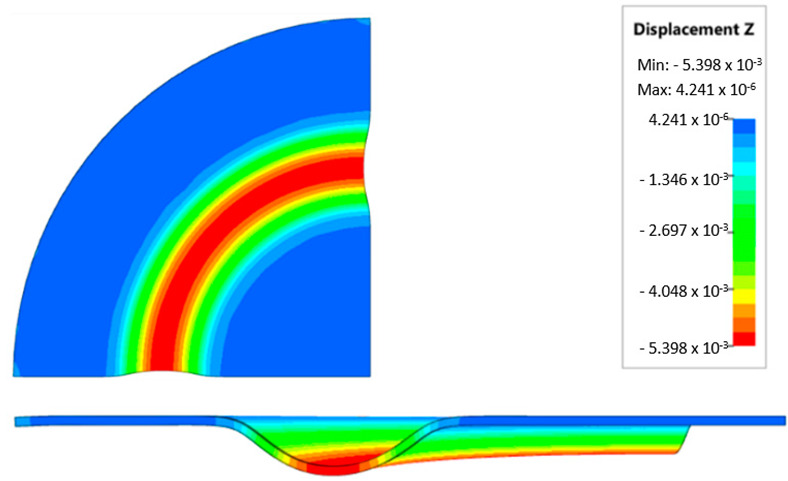	5.398 mm
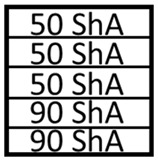	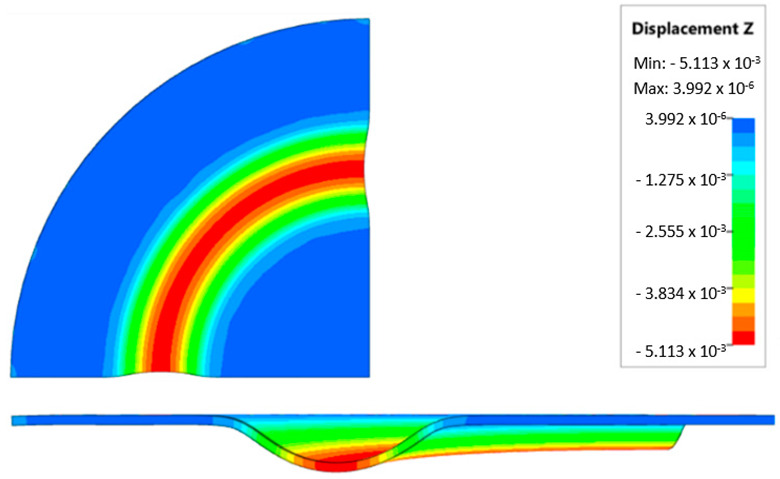	5.113 mm

**Table 8:** Results of forming cups with a pressure of 1.000 kN.

Elastomeric Punch—Hardness	Formed Sheet—Image	Formed Sheet—3D Scan	Maximum Cup Depth
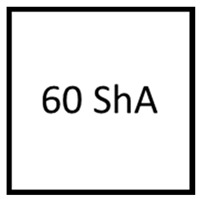	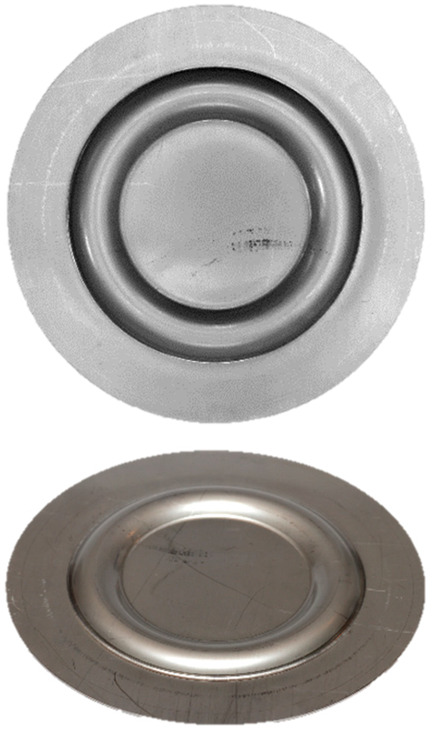	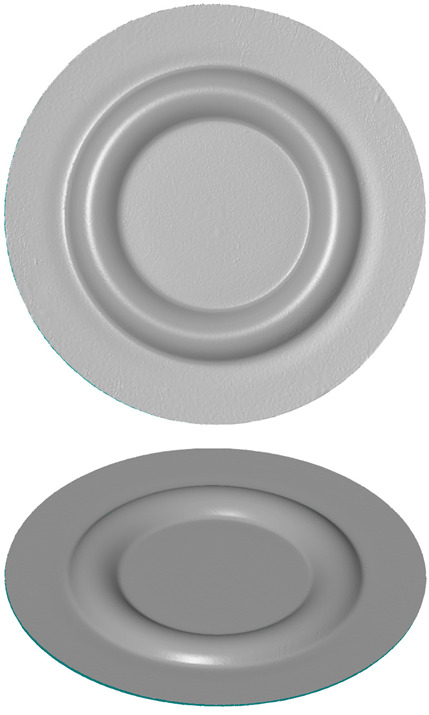	5.37 mm
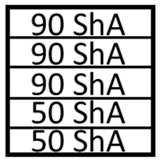	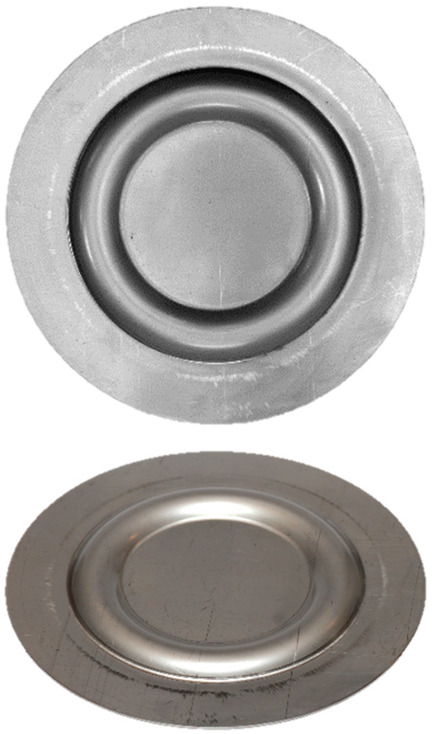	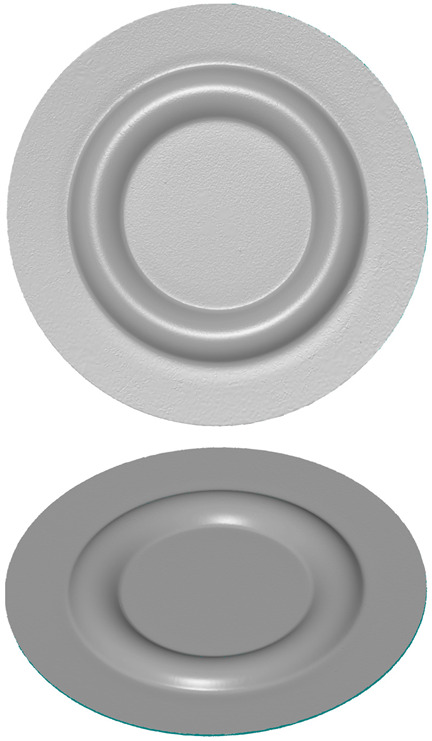	5.56 mm

## Data Availability

Data are contained in the article.
